# Targeting Tyrosine kinases in Renal Cell Carcinoma: “New Bullets against Old Guys”

**DOI:** 10.3390/ijms20081901

**Published:** 2019-04-17

**Authors:** Teresa Alonso-Gordoa, María Laura García-Bermejo, Enrique Grande, Pilar Garrido, Alfredo Carrato, Javier Molina-Cerrillo

**Affiliations:** 1 Medical Oncology Department, The Ramón y Cajal Health Research Institute (IRYCIS), CIBERONC, Alcalá University, University Hospital Ramon y Cajal, 28034 Madrid, Spain; pilargarridol@gmail.com; 2 Biomarkers and Therapeutic Targets Group and Core Facility, Ramón y Cajal Research Institute, (IRYCIS), 28034 Madrid, Spain; garciabermejo@gmail.com; 3 Medical Oncology Department, MD Anderson Cancer Center, 28034 Madrid, Spain; egrande@oncomadrid.com; 4 Medical Oncology Department, Ramón y Cajal Health Research Institute (IRYCIS). CIBERONC, Alcalá University, University Hospital Ramon y Cajal, 28034 Madrid, Spain; acarrato@telefonica.net

**Keywords:** Tyrosine kinase, Kidney cancer, Vascular endothelial growth factor receptor (VEGFR), Platelet Derived Growth Factor Receptor (PDGFR), Tyrosine-Protein Kinase Met (MET), Axl, Fibroblast Growth factor Receptor (FGFR)

## Abstract

Clear cell renal cell carcinoma (ccRCC) is the seventh most frequently diagnosed tumor in adults in Europe and represents approximately 2.5% of cancer deaths. The molecular biology underlying renal cell carcinoma (RCC) development and progression has been a key milestone in the management of this type of tumor. The discovery of Von Hippel Lindau (*VHL*) gene alterations that arouse in 50% of ccRCC patients, leads the identification of an intracellular accumulation of HIF and, consequently an increase of VEGFR expression. This change in cell biology represents a new paradigm in the treatment of metastatic renal cancer by targeting angiogenesis. Currently, there are multiple therapeutic drugs available for advanced disease, including therapies against VEGFR with successful results in patients´ survival. Other tyrosine kinases’ pathways, including PDGFR, Axl or MET have emerged as key signaling pathways involved in RCC biology. Indeed, promising new drugs targeting those tyrosine kinases have exhibited outstanding efficacy. In this review we aim to present an overview of the central role of these tyrosine kinases’ activities in relevant biological processes for kidney cancer and their usefulness in RCC targeted therapy development. In the immunotherapy era, angiogenesis is still an “old guy” that the medical community is trying to fight using “new bullets”.

## 1. Introduction

Kidney cancer represents the third tumor of the urinary tract most frequently diagnosed in adults. In Europe, the incidence of clear cell renal cell carcinoma (ccRCC) accounts for 84,000 new cases per year with a mortality rate of about 35,000 patients in 2012 [1]. The clear cell subtype is the most common one representing approximately 85% to 90% of all renal cancer diagnosis [2]. The mortality rate varies significantly throughout different regions and is associated to the availability of more sophisticated diagnostic techniques, multidisciplinary teams and effective systemic drugs. In fact, in countries from Northern and Eastern Europe, Australia and US, the mortality tends to stabilize or even decline [3]. Additionally, the incidence of kidney cancer is greater in men than women (ratio of 2:1) [4] and it is usually diagnosed around the sixth decade of life (median age around 67 years). 

In recent years, kidney cancer diagnosis has increased among early stages and only 20% of patients present metastases at tumor diagnosis. Despite radical treatment for localized disease, around 20% to 40% of patients relapse within five years [5,6].

Until 2006, the treatment of the metastatic disease was based on cytokines that had an unfavorable safety profile and achieved poor efficacy results with a progression-free survival (PFS) of around five months and overall survival (OS) of around 21 months [7].

The initial discovery of the role that angiogenesis has in kidney cancer development and progression has led the research of new agents that target key players of this step. The efficacy demonstrated by sunitinib, a tyrosine kinase inhibitor (TKI), targeting mainly the vascular endothelial growth factor receptor (VEGFR) and platelet-derived growth factor receptor (PDGFR), has been able to demonstrate a change in the natural history of metastatic kidney cancer and, quickly, prompted the use of TKI in the therapeutic algorithm of metastatic ccRCC patients. Sunitinib in the first line setting achieved an increase in the median PFS to up to 11 months and OS to approximately 26 months [8].

Since then and until now, more drugs have been developed and approved by the regulatory agencies for the treatment of metastatic ccRCC including tyrosine kinase inhibition strategies alone or in combination with other therapeutic approaches. For example, the inhibition of the mTORC complex by everolimus or temsirolimus presented benefits in a selected patients´ population and new immunotherapy strategies, such as nivolumab or its combination with ipilimumab, presented positive results in terms of efficacy and survival post VEGF-therapy and in the first line setting respectively [9,10].

The aim of this review is to provide an updated-global view of the role of tyrosine kinases in metastatic kidney cancer and to understand the mechanism of action of the different TKIs act over them to achieve a clinical benefit in patients suffering from this disease. 

## 2. Molecular Biology of Kidney Cancer

There are several genes involved in the development of both sporadic and hereditary renal cancer. Among them, VHL is the most important gene, standing at around 50% of ccRCC [3]. Mutations in the PTEN/PI3K/mTOR axis lead to permanent activation of the mTORC complex, underlying ccRCC development and progression.

Epigenetic alterations are also critical for ccRCC development, therefore genes involved in chromatin remodeling, such as PBRM1, BAP1, SETD2, and KDM5C, also play a role in this setting of the disease.

These gene alterations and the phenotypic results within the biology of kidney cancer cells are key to understanding the exact mechanisms responsible for ccRCC development and progression and the underlying mode of action of the treatment used for these patients in daily practice [11,12].

### 2.1. Von Hippel Lindau (VHL)

VHL is located in the short arm of chromosome 3 (3p25) and plays a central role in the development of renal cancer as a tumor suppressor gene. Somatic alterations (mutations or epigenetic alterations) have been described in a large number of sporadic cases of ccRCC [13]. Its germline alteration leads to the hereditary syndrome of Von Hippel-Lindau characterized by the development of renal cysts and ccRCC, among other clinical manifestations [14].

The protein derived from the VHL gene forms a complex with elongin B, cullin 2 and elongin C, as well as the ubiquitination complex E3 in its α domain [15]. This complex has the main function of ubiquitination after HIF hydroxylation, HIF-2α and HIF-1α. This mechanism occurs normally in normoxic conditions and avoids the accumulation of intranuclear HIF-1α and HIF-2α [16]. This process in normoxia is carried out by the presence of HIF prolyl hydroxylase (PHD), which owns three subunits, and is in charge of the critical enzymatic step of HIF 1α/2α proline residue hydroxylation. This step requires the presence of O_2_ and Fe^+2^ as co-factors and allows the binding of the VHL active complex to HIF α through its β domain, its ubiquitination and subsequent proteasome degradation [17,18].

Under hypoxic conditions, PHD is unable to hydroxylate the proline residue of HIF due to the lack of O_2_ and, therefore, cannot form the ubiquitination complex, leading to HIF 1α/2α accumulation in cytoplasm [19,20]. This accumulation allows HIF dimerization (HIFα and HIFβ) and subsequent translocation to the nucleus and transcriptional regulation of all its targets. These targets include VEGF/PDGF and also MET and Axl, as well as other intracellular signaling proteins such as those involved in the mTORC complex [21,22].

Based on this, the initial targeted treatment strategies focused on antiangiogenic drugs, which counteract the over-accumulation of intracellular HIF and the subsequent overexpression of VEGF or PDGF by blocking their receptors VEGFR and PDGFR signaling, among others. Drugs against the mTORC complex in monotherapy have shown modest activity and are currently considered in pretreated patients or patients with poor memorial Sloan-Kettering cancer center (MSKCC) prognosis criteria (Appendix A) or different histological subtypes from clear cell (such as cromophobe) because they have been outperformed in efficacy and survival by other therapeutic strategies [22].

### 2.2. Other Genetic Alterations

There are other genes involved in the development of kidney cancer. Of special interest are those related to chromatin remodeling and histone acetylation processes, such as BAP1, PBRM1 and SETD2, acting as tumor suppressor genes [4]. Interestingly, these three genes are also located in the short arm of chromosome 3, specifically in the 3p21 region, very close to VHL. Thus, the loss of the short arm of chromosome 3, a frequent event in renal cancer, leads to the loss of VHL, but also BAP1, PBRM1 and SETD2 and, therefore, the loss of important tumor suppressor genes [23].

The alterations in these chromatin remodeling genes and the clinical impact in renal cell carcinoma (RCC) is not entirely clear. It seems that patients with these gene alterations develop more aggressive diseases, with highly heterogeneous tumors and, from a clinical point of view, those patients exhibit worse survival and response to oncological treatments [24,25].

MET is involved in different processes in RCC. On the one hand, the activating mutations have been described in the tyrosine kinase domain involved in the development of papillary subtypes of renal cancer [26]. On the other hand, MET and Axl are direct targets of HIF, and the accumulation of HIF induces the accumulation, not only of VEGF, but, also of MET and Axl [27]. Moreover, MET and Axl are involved in angiogenic resistance mechanisms commonly used in the RCC, such as sunitinib. Under therapeutic pressure for a long period of time with sunitinib, kidney tumors could eventually overexpress MET and Axl signaling pathways as an escape mechanism [28]. Therefore, MET and Axl are involved in different moments of the disease, justifying the development of drugs against them.

## 3. Tyrosine Kinases and Coupled Intracellular Signaling Involved in RCC

### 3.1. Vascular Endothelial Growth factor Receptor (VEGFR)

**Figure 1:**
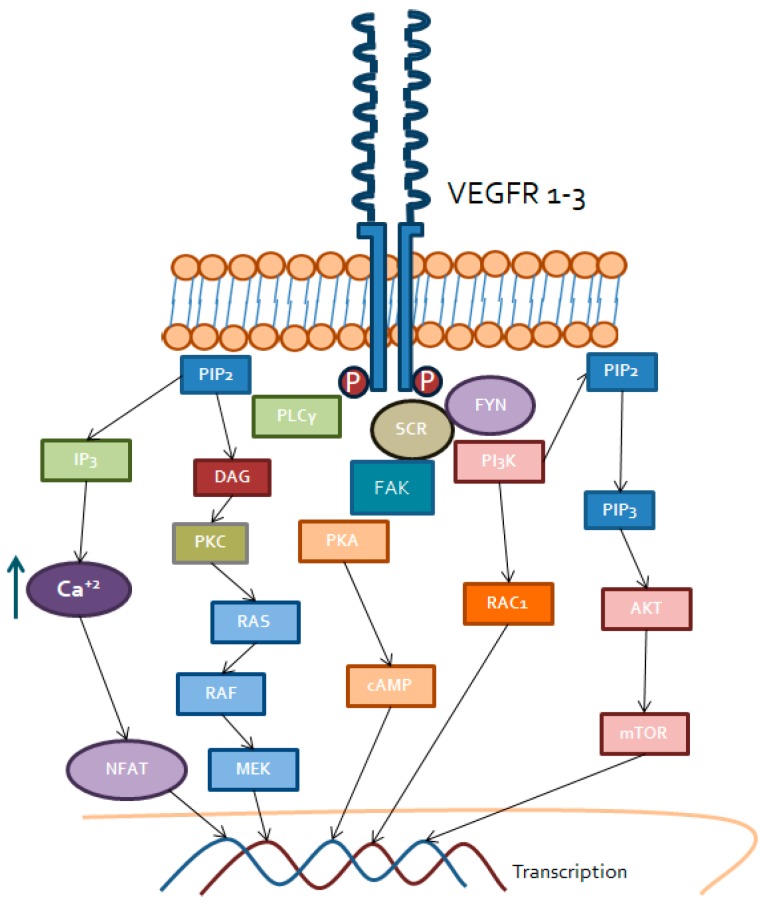
Intracellular signaling triggered by VEGF receptors. Specific transmembrane receptor tyrosine kinase for VEGF, i.e., VEGFR 1–3, recruit PLCγ, Src and FYN in order to signal mainly through DAG receptors (PKC), PKA, Rac1, MEK and Akt/mTOR. Transcription factors activated by these signaling, including NFAT or AP1, will finally regulate gene expression associated to VEGFR/VEGF binding. Increased levels of intracellular calcium also linked to this binding will contribute to PKC and NFAT activation, among others.

Vascular endothelial growth factor receptors (Figure 1) represent a family of three receptors: VEGFR1, VEGFR2 and VEGFR3. The most important one, from a biologically point of view, is VEGFR2, which is the main receptor for VEGF in the vascular endothelium, regulating different intracellular processes [29].

All members of the VEGFR family have the same structure, consisting in a seven extracellular immunoglobulin-like domain, a transmembrane domain and an intracellular tyrosine kinase domain. They are mainly regulated by their soluble ligands, among which are VEGF A/B/C/D, placenta growth factor (PlGF), parapoxvirus VEGFE, snake venom VEGFF and neuropilins NRP1 and NRP2 [30,31]. However, there is another way of VEGFR activation, mainly VEGFR2, by non-canonical activation mechanisms that would explain other functions that are attributed to this receptor. These non-canonical ways of activation would include mechanical stimulation, with the formation of mechanosensory complexes between VEGFR2 or vascular endothelial cadherin and platelet endothelial cell adhesion molecule 1 (PECAM1). When the complex is built, it is able to regulate the endothelial nitric oxide synthase (eNOS) [30,32]. In addition, VEGFR2 can interact with galectins, mainly galectin 3, expressed ubiquitously on the cell surface and with low density lipoproteins (LDL) [33].

As we have already presented, the VEGF ligands are able to bind their membrane receptors, but with different affinities. VEGFA is able to bind all three receptors, VEGFC and D do the same, but mainly to VEGFR2 and VEGFR3, and VEGFB and PIGF have greater affinity for VEGFR1 binding [34,35].

Once the ligand is bound, stable VEGFR dimers are generated to trigger activation of the tyrosine kinase domains and initiate intracellular signaling. The most important dimers in the vascular endothelium are the VEGFR2 homodimers. However, heterodimers between VEGFR1-VEGFR2 and VEGFR2-VEGFR3 can also be present. The first one exists in atherosclerotic lesions and in the embryonic endothelium and the second one, mainly in the lymphatic endothelium. Those heterodimers are known to be involved in cancer-related processes, but by not yet clearly defined paths [30,36]. Intracellular signaling triggered involves PI3K-AKT-mTOR and phospholipase Cγ (PLCγ) pathways.

#### 3.1.1. PI3K/AKT/mTOR

Even after phosphorylation of the tyrosine kinase domain of VEGFR2, PI3K activation requires cross-activation of the Src kinase family (Src, YES and FYN) [37]. This kinase family is in charge of cytoskeletal dynamics, intracellular junctions’ maintenance and vascular permeability regulation by phosphorylation of focal adhesion kinase (FAK). Once Src has activated PI3K, it is able to phosphorylate the phosphatidyl inositol biphosphate (PIP2) of the cell membrane into the phosphatidyl inositol triphosphate (PIP3) [38]. This, in turn, can activate the AKT complex, mainly AKT1 and, finally, regulate cell functions related to survival and proliferation by the interaction of AKT with the mTOR complex [39]. In addition, this pathway regulates the activity of eNOS, a key molecule in endothelial permeability and vasodilation, as well as cell migration by activating GTPases, such as RHO and RAC1 [40,41].

#### 3.1.2. Phospholipase C (PLCγ) Pathway

In a similar way, after activation of the tyrosine kinase domain of VEGFR2, direct phosphorylation of PLCγ occurs. This kinase has PIP2 as substrate, transforming it into inositol triphosphate (IP3) and diacylglycerol (DAG). Both of them are able to stimulate the release of calcium by the endoplasmic reticulum into the cytosol, causing several effects. On the one hand, it activates protein kinase C (PKC) dependent on calcium, and other DAG receptors such as chimerins, enhancing the RAS-RAF-ERK pathway [42,43]. This signaling cascade regulates fundamental endothelial processes such proliferation, survival and migration. On the other hand, the increase of intracellular calcium renders in activation of proteins sensitive to ion changes in the cytoplasm, such as calmodulin and calcineurin. Calcineurin has serine-threonine phosphatase activity and is able to remove phosphate of the nuclear factor of activated T cell (NFAT), thus allowing the translocation of NFAT into the nucleus, where it regulates the expression of kinases dependent on cyclins, such as CDK2 or CDK4, proteins involved in cell proliferation or cell survival processes [44,45,46].

### 3.2. Platelet Derived Growth Factor Receptor (PDGFR)

Platelet derived growth factor receptors consist of two members: PDGFRα and PDGFRβ. These receptors are composed of five extracellular immunoglobulin-like domains, a transmembrane domain and an intracellular tyrosine kinase domain in the C-terminal region [47]. Their signaling is fundamental in embryonic development, as well as in wound repair. Moreover, there is an increased interest about their involvement in pathological processes, such as cancer, due to their role in tumor microenvironment maintenance [48].

For their tyrosine kinase domain activation, these receptors need a ligand binding to achieve subsequent stabilization and dimerization. PDGFs are synthesized as pre-proteins and require the activity of proteases to expose their mature and active form. There are four dimeric isoforms, as PDGF homodimer: PDGF AA/BB/CC/DD and one heterodimeric isoform constituted by PDGF AB [48].

PDGFs are synthesized by different cell types, mainly mesenchymal cells, endothelial cells, some epithelial strains and myeloid cells, such as macrophages or platelets. Moreover, all these cells also express PDGFRα and/or PDGFRβ, so that PDGFs usually lead to autocrine regulation loops [49,50].

Similar to other tyrosine kinase receptors, not all PDGF subtypes result in the formation of the same dimers. PDGF-BB, PDGF-CC, PDGF-DD and PDGF-AB lead to the formation of the dimers PDGFRα/PDGFRβ, PDGF-AA, PDGF-AB, PDGF-BB and PDGF-CC do the same with the dimers PDGFRα/PDGFRα and PDGF-BB and PDGF-DD lead to PDGFRβ/PDGFRβ [51,52].

Once the intracellular tyrosine kinase domain is activated, PDGFR is able to regulate several signaling pathways. On the one hand, PI3K is attracted to the receptor and later activated. PI3K activation transforms PIP2 into PIP3, which is able to bind and activate AKT and, therefore, trigger the mTOR complex to regulate genes related to cell survival [53]. In addition, the tyrosine kinase domain is also able to attract PLCγ, which transforms PIP2 into IP3 and DAG, increasing intracellular calcium and activating PKC to ultimately regulate cell growth and survival [54]. On the other hand, after activation of the tyrosine kinase domain, an interaction with the Nck adapter protein occurs and C-Jun N-terminal protein-serine/threonine kinase (JNK) is activated by the RAS and MAPK pathway. Cell proliferation and survival processes are consequently regulated after PDGFR activation [55].

Active PDGFR is also able to recruit Src and, together with Gbr2 and Sos, is able to phosphorylate RAS-GDP to RAS-GTP. Consequently, there is an activation of relevant oncogenic signaling pathways via RAS/RAF/ERK, which mainly regulate cell proliferation-related genes [56].

It should be noted that PDGFR is intimately linked and related to KIT and FLT3, which explains why TKIs with activity against all these targets are effective in the treatment of renal cancer [57].

### 3.3. Tyrosine-Protein Kinase Met (MET)

**Figure 2:**
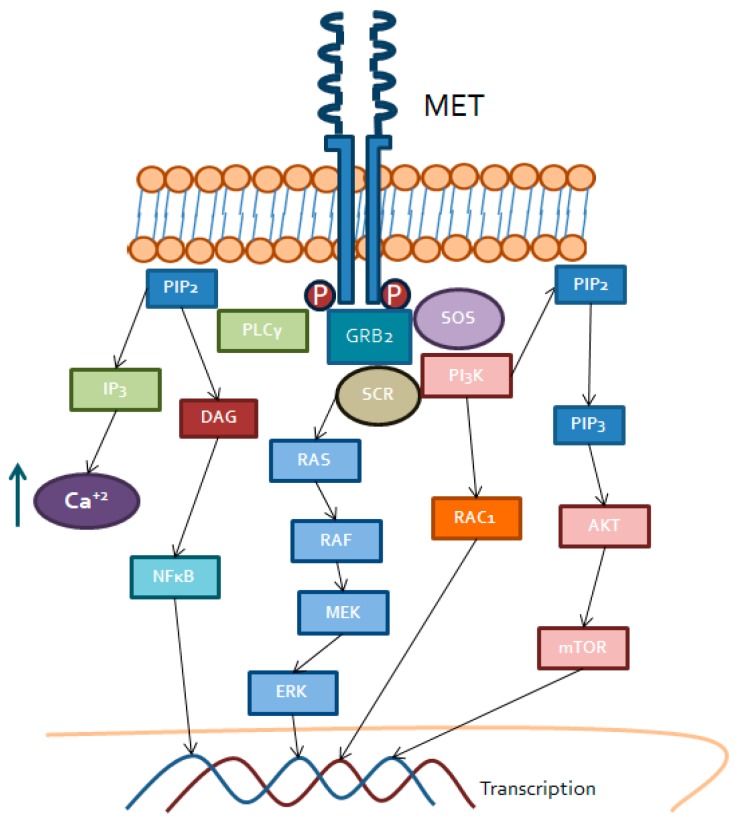
MET triggered intracellular signaling. Binding of HGF to MET transmembrane receptor tyrosine kinase recruit PLCγ, GRB2 and SOS, which activate DAG receptors (PKC), RAS/RAF/MEK, PI3K/RAC and Akt/mTOR. These kinases lead to activation of transcription factors including NFκB or AP1 for gene expression.

Tyrosine-protein kinase MET (Figure 2) or the hepatocyte growth factor receptor (HGFR) is a membrane receptor involved in multiple biological routes. Initially, it is synthesized as a single chain and, subsequently, through post-transcriptional modulation, the α and β subunit result in the mature receptor as a disulfide-linked heterodimer [58].

MET has an extracellular domain with four subdomains: immunoglobulin-like for ligand binding, a membrane anchoring domain and an intracellular domain, at the C-terminal end of the protein where the tyrosine kinase region is located, with different binding sites for substrates and ligands [59].

The known ligand of MET is the hepatocyte growth factor (HGF). Mainly by mesenchymal cells, it is secreted into the medium as an inactive form and, by the activity of extracellular proteases, it turns into a mature protein. At this time, HGF is able to perform its paracrine function on epithelial cells where it is usually expressed [60]. HGF regulates cell proliferation, motility, survival and growth functions of endothelial cells and also the hydrolysis of extracellular matrix proteins. The latest function justifies the key role of MET in embryonic development, as well as in other pathological processes, such as cancer [61].

Once HGF binds MET, it produces oligomerization of the receptor and the subsequent activation of the tyrosine kinase domain to exert the transcriptional regulation [62]. The expression of HGF and MET, as well as the regulation of this pathway, can also be affected by interleukins, such as IL-1and IL-6 or hypoxia-related factors, such as HIF 1α/2α, tumor necrosis factor-α (TNFα) or some subtypes of FGF, such as bFGF [63].

Once the tyrosine kinase domain is activated, several molecular signaling pathways are stimulated. Those pathways are mainly PI3K/AKT and RAS/MAPK/ERK together with other auxiliary proteins, such as Sos, Gbr2 and including the activation of Src, along with NFκB regulation [64,65]. All these pathways regulate, therefore, cell proliferation, survival, angiogenesis, motility and epithelial to mesenchymal transition (EMT).

The HGF/MET pathway interacts with other cell pathways that can reinforce its activation. In fact, in this sense, several escape mechanisms to various drugs have been described. Interaction with EGFR/HER2 mainly occurs because EGFR can activate Src-dependent MET in a ligand-independent manner [61]. HGF/MET also interacts with the VEGFR2 pathway, mainly by increasing VEFGA, and the NOTCH or βcatenin and WNT pathways, which partly explains the role of this receptor in EMT, angiogenesis and tumor growth and migration [66,67].

### 3.4. Axl

Axl is part of the TAM receptors family, constituted by three members: Tyro3, Mer and Axl itself. The TAM family is expressed mainly in the liver, central nervous system, platelets and in some cells of the innate immune system and the vascular endothelium [68]. All of them have different physiological functions, but they have in common their important relationship with cell proliferation and motility in cancer cells. Axl is also involved in angiogenesis and hematopoiesis, unlike Mer that is involved in immunosuppressive cell mechanisms [69,70].

The Axl structure is similar to other tyrosine kinase receptors. It has an extracellular domain with two immunoglobulin-like ligand binding domains, a transmembrane region and an intracellular region where the tyrosine kinase dominion is located [71].

Growth arrest-specific protein 6 (Gas6) is the most important ligand of Axl, but it is not the only one, since tubby-like protein 1 (TULP-1) can also enhance the activation of this receptor. Usually, the binding of Gas6 with Axl occurs with two molecules of Gas6 favoring the dimerization of two Axl proteins, with the subsequent autophosphorylation of the tyrosine residues in the tyrosine kinase domain and the activation of the dependent intracellular signaling cascades [69,72].

Although the most frequent way for Axl activation is through Gas6, there are other alternative manners. Axl can also be activated independently from the receptor: Under oxidative stress conditions, by interaction with another Axl receptor in the same cell or with the one of a nearby cell or by hetero-dimerization with other receptor families, such as VEGFR1 or other members of the TAM family [73].

Once tyrosine kinase domain is activated, Axl is able to exert its activity through different signaling pathways. It is able to recruit PI3K/AKT/mTOR that regulates cell survival through the expression of NFκB. Furthermore, it acts over the RAS/RAF/ERK and PI3K/RAC pathways to regulate proliferation and cell motility. In addition, it affects other membrane receptors such as MET, VEGFR or EGFR and participates in EMT processes promoting invasiveness [74,75].

This crosstalk of Axl with other signaling pathways has been considered a mechanism of resistance against drugs targeting molecules such as VEGFR, EGFR, BRAF or even ALK. Overall, Axl in kidney cancer is involved in angiogenesis and in resistance mechanisms over drugs against VEGFR [76,77,78].

### 3.5. Fibroblast Growth factor Receptor (FGFR)

FGFR is a family of membrane receptors consisting of five components [79]. FGFR1-4 has tyrosine kinase activity, while FGFR5, although it seems to have affinity for the FGFs, has no intracellular tyrosine kinase domain [80].

This family of receptors binds to FGF ligands that are secreted into the medium in order to be activated and fulfill their intracellular functions. The FGF ligands are divided into seven subfamilies according to their origin and structure [81,82,83]:

- FGF1: Constituted by FGF1, is also known as aFGF or FGF acid and FGF2 or bFGF (basic FGF);
- FGF4: Constituted by FGF4, FGF5 and FGF6;
- FGF7: Constituted by FGF3, FG7, FGF10 and FGF22;
- FGF9: Constituted by FGF9, FGF16 and FGF20;
- FGF8: Constituted by FGF8, FGF17 and FGF18.

These forms are considered canonical or paracrine and recruit heparin or heparan sulfate as a cofactor to activate FGFR.

- FGF11: Constituted by FGF11, FGF12, FGF13 and FGF14.

The FGF11 family is also known as intracellular FGFs and use ion channels as activating cofactors.

- FGF15/19: Constituted by FGF15/19, FGF21 and FGF23.

This subfamily is also known as the endocrine FGFs and they use proteins from the Klotho family as cofactors: FGF 15/19 and FGF21 mainly need βKlotho and FGF23 needs αKlotho.

All FGFs are not expressed in the same spatial or temporal framework, in fact, some of them are only relevant in embryonic development [84]. However, others play an important role in adult life: Activating FGFRs. These are expressed by different types of tissues and regulate cell proliferation, migration, survival and differentiation. In addition, they also regulate other growth factor receptors such as EGFR, VEGF or HGF, so a role in angiogenesis and inflammation has been suggested for FGFR [85,86].

FGFR exhibits a structure including three immunoglobulin-like extracellular domains that bind to FGFs, a transmembrane domain and an intracellular tyrosine kinase domain [87]. Once FGF binds, FGFR dimerizes causing autophosphorylation of the tyrosine residues in the tyrosine kinase domain and triggers the intracellular signaling cascade. FGFR is able to recruit Src and its effectors, to finally activate RAS/ERK and also, directly phosphorylates FGFR substrate 2 (FRS2) that is able to activate the PI3K/AKT pathway. In addition, the activation of FGFR leads the activation of PLCγ and PKC to activate MAPK [83,88,89].

## 4. Tyrosine Kinases and Coupled Intracellular Signaling Involved in RCC

VEGF-targeted agents have been approved for the treatment of metastatic ccRCC and the sequence of these drugs keeps antitumor activity considering the differences between kinases specificities [90]. This benefit along the different TKIs administered sequentially has been demonstrated even in clinical trials with positive results in terms of survival. For patients´ prognosis stratification we still consider clinical values and two main clinical prognostic scores that have been validated and currently used in clinical practice and clinical trials: The MSKCC and international metastatic RCC database consortium (IMDC) prognostic risk criteria (Appendix A and Appendix B) [91,92].

### 4.1. Sunitinib

Sunitinib was the first VEGFR targeted drug to change the natural history of metastatic RCC from the first line setting compared with the standard of care at that time (cytokine-based treatment). It is an antiangiogenic drug that has antitumor and antiangiogenic activity through the inhibition of PDGFRα, PDGFRβ, VEGFR1-3, KIT, FLT3, CSF-1R and RET. This drug is administered at a dose of 50 mg every day during four weeks with two additional weeks of rest. The clinical development of sunitinib in kidney cancer was based on the three objective tumor responses identified in the four patients with metastatic RCC included in a phase I trial for solid tumor malignancies [93]. Consequently, two main phase II trials were conducted in patients previously treated with cytokines and the promising results were supported by a 34% to 40% of partial responses and 8.3 months to 8.7 months of median PFS. Definitely, the phase III trial recruited 750 patients randomized to sunitinib or interferon-α (7) (Table 1). The results of this pivotal trial showed a benefit in median PFS of 11 months in the sunitinib group and five months in the interferon-α group (HR 0.42 (95% CI, 0.32 to 0.54; *p* < 0.001)). Based on the better tolerability of sunitinib compared with the standard of care at that time and the survival benefit, the Food and Drug Administration (FDA) and European Medicines Agency (EMA) approved sunitinib for the upfront treatment of patients with metastatic RCC in 2006. The results of the previously mentioned clinical trials were validated in expanded access programs conducted within the following years. Those data confirmed the activity of sunitinib in a group of patients with clinical characteristics more similar to the clinical practice [94].

The safety profile of sunitinib forces to frequent dose reductions, but tolerability was improved by the research of alternative schedules to the standard four weeks on/two weeks off with two weeks on/one week off. This schedule was based on the data obtained in phase I trials [95] with pharmacokinetic studies evaluating the relationship between a dosing regimen and the concentrations achieved in patients with solid tumors. The results suggested that the exposure to sunitinib and its active metabolite of the 2/1 scheme were comparable to that of the 4/2 scheme due to the ability of sunitinib and its metabolite of reaching the state of equilibrium between seven to 14 days and 14 days, respectively. We also have information obtained from the pharmacological analysis carried out in six studies with sunitinib administered to patients with solid tumors, including GIST and metastatic RCC. Those studies suggested that those patients who reached a greater exposure to the drug, measured by the area under the curve (AUC), have better survival outcomes and tumor responses. Those treatment regimens were evaluated in retrospective studies and phase II prospective trials confirming the activity of the alternative schedule with a significant benefit in safety and tolerability [96], mainly in hand-foot syndrome and asthenia, two adverse events that clearly have an impact in quality of life deterioration.

### 4.2. Pazopanib

Pazopanib shows activity against VEGFR1-3, PDGFRα and β, FGFR and cKIT. This potential antiangiogenic activity was the rational for the recruitment of 435 patients in a phase III trial of pazopanib 800 mg daily compared with placebo. Patients were treatment-naïve and refractory to previous cytokine-based therapy [97]. The results showed an overall response rate (ORR) of 30% versus 3% for pazopanib compared with the placebo, respectively. The median PFS for treatment-naïve patients were 11.1 versus 2.8 months and for cytokine-refractory patients of 7.4 versus 4.2 months, for pazopanib versus placebo, respectively. In 2014, the results of the non-inferiority COMPARZ trial of sunitinib versus pazopanib were published [98] (Table 1). The study population included 927 patients from the original population and 183 from a phase II substudy conducted with Asian patients. The analysis performed with a number of events of 659 showed that pazopanib was not inferior to sunitinib in the median PFS fulfilling the pre-established margin of non-inferiority for a HR ≥ 1.25. The median PFS was 8.4 months for pazopanib arm (95% CI, 8.3 to 10.9) and 9.5 months for sunitinib arm (95% CI, 8.3 to 11.1) with a HR = 1.05 (95% CI, 0.90 to 1.22). Adverse events that were more frequently reported with pazopanib were a change in hair color, alopecia and weight loss and more severe adverse events were liver enzymes and bilirubin increase. On the contrary, more frequently reported adverse events with sunitinib were, of any grade, fatigue, hand and foot syndrome (HFS), mucosal inflammation, stomatitis, hypothyroidism, dysgeusia, dyspepsia, epistaxis, hematologic alterations and ionic disturbances. Based on the results of this trial, pazopanib also became a standard of care in the first line setting of patients with metastatic ccRCC.

### 4.3. Tivozanib

Tivozanib has reached the EU approval in the first line setting of metastatic ccRCC based on the results of the TIVO-1 trial. Tivozanib selectively inhibits VEGFR 1–3 at picomolar concentrations so, even if it is considered a multitargeted tyrosine kinase, it has the advantage of inducing less off-target adverse events than other TKIs. The maximum tolerated dose obtained from the phase I trial was 1.5 mg/day during three weeks with one week off treatment.

The TIVO-1 trial included patients not previously treated with a VEGFR inhibitor or an mTOR inhibitor [99] (Table 1). However, patients could have received one previous treatment line of cytokine-based therapy. The primary endpoint was PFS by independent radiological review and patients were offered to crossover to tivozanib at disease progression. The first cut off, in December 2011, showed a benefit in the median PFS of 11.9 months for tivozanib versus 9.1 months for sorafenib (HR: 0.797; 95% CI: 0.639–0.993; *p* = 0.042). Subgroup analysis showed a greater benefit from tivozanib in the favorable IMDC risk group (HR: 0.387; 95% CI: 0.200–0.748; *p* = 0.003), favorable MSKCC risk group (HR: 0.590; 95% CI: 0.378–0.921; *p* = 0.018) and treatment naïve patients (HR: 0.756; 95% CI: 0.580–0.985; *p* = 0.037). Although no significant OS benefit was observed, only 26% of patients in the tivozanib group received any second line therapy compared with 65% (of those, 92.5% received tivozanib) in the sorafenib group due to the different approved drugs available in the countries participating in the TIVO-1. For example, in Central and Eastern Europe 23% in the tivozanib arm (*N* = 229) versus 64% in the sorafenib arm (*N* = 228) received next-line therapy and in North America and Western Europe 59% in the tivozanib arm (*N* = 22) versus 78% in the sorafenib arm (*N* = 18) received next-line therapy. In this sense, this trial gives information to physicians about the sequential treatment of two TKIs (sorafenib-tivozanib) compared with a TKI monotherapy (tivozanib). Moreover, patients enrolled in the sorafenib arm were recruited in a phase 2 crossover trial to tivozanib (*N* = 161). Those patients achieved a median PFS of 11 months and median OS of 21.6 months in second line treatment with tivozanib preceded by sorafenib [100]. 

The activity of tivozanib has also been evaluated in the TIVO-3 trial with patients previously treated with at least two or three treatment lines (containing ≥ one VEGFR inhibitor, Table 2). The interim analysis of this trial has been presented at the 2019 Genitourinary Cancers Symposium and the final analysis is expected to be due after a cut-off date on August 2019 [103]. The population included in this study had received prior two and three treatment lines in 62% and 38% of patients, respectively. Previous treatment with a PD-1 inhibitor was administered in 27% of patients. The primary endpoint was achieved with a median PFS of 5.6 months for tivozanib arm versus 3.9 months for sorafenib arm (HR = 0.73 (95%CI 0.56–0.94) *p* = 0.0165). Data on OS, though still immature, were presented and no significant benefit of tivozanib over sorafenib was observed.

### 4.4. Axitinib

The FDA and EMA approval of axitinib for the treatment of patients with kidney cancer was based on the data of the phase III trial AXIS in 2012 (Table 2). Axitinib is also characterized by the specific inhibition of VEGFR 1-3. The AXIS trial included 723 patients with metastatic ccRCC that had received only one previous treatment (sunitinib, bevacizumab plus IFNα, temsirolimus or cytokines) [104]. Patients were randomized to receive treatment with axitinib 5 mg/12 h versus sorafenib 400 mg/12 h. After 477 events, the median PFS was 6.7 months for axitinib versus 4.7 months for sorafenib (HR 0.665, IC95% 0.54–0.812; *p* < 0.0001). However, this trial did not reach the secondary endpoint of OS (20.1 months vs. 19.2 months (HR 0.97; IC95% 0.8–1.17; *p* = 0.37)) [105].

Particularly from axitinib, relevant information is available concerning pharmacokinetic research evaluating the association between the dose administered, adverse events appearance and efficacy of the proper TKI. In this sense, from phase I trials that identified a direct relationship of the dose and the pharmacokinetic analysis, many data suggested that an increase in the dose could improve the activity of the drug in those patients with a good tolerability at a dose of 5 mg/12 h. In order to evaluate this therapeutic strategy of individualising the axitinib dose in a prospective manner, a phase II trial was conducted in patients not previously treated [106]. All patients initiated treatment with axitinib 5 mg/12 h during four weeks and, at that time, those with adequate blood pressure control, no other severe axitinib-related toxicities and no dose reduction requirement were randomized to axitinib versus placebo dose titration. The results of this trial showed that those patients in the axitinib dose titration reached a greater exposure of the drug and this was associated with an increase in ORR (54% versus 34%). However, no significant differences were observed in survival outcomes (HR = 0.84; *p* = 0.24).

### 4.5. Cabozantinib

The rational for cabozantinib treatment in kidney cancer is based, not only in the inhibition of VEGFR, but also by the activity over MET and Axl. 

A phase I clinical trial evaluated the tolerability of this drug and the combination with rosiglitazone, a substrate for CYP2C8, to evaluate the drug-drug interaction [107]. The dose of cabozantinib was 140 mg daily. Twenty-five previously treated patients with metastatic ccRCC were included. Eighty percent of patients required dose reduction and the most frequent grade ≥ 3 adverse events reported were fatigue (20%) and diarrhea (12%). From this initial research, the ability of cabozantinib to achieve a tumor control growth has been observed. Only one patient was refractory to cabozantinib, seven patients demonstrated a partial response and 13 patients had stable disease according to the Response evaluation criteria in solid tumors (RECIST) criteria. Those promising results led the development of the phase III METEOR trial that randomized 375 patients previously treated with at least one VEGFR inhibitor to cabozantinib 60 mg daily vs. everolimus 10 mg daily [108] (Table 2). The coprimary endpoint of PFS was achieved with a median PFS of 7.4 months in the cabozantinib arm (95% CI 5.6–9.1) versus 3.8 months in the everolimus arm (95%CI 3.7–5.4) (HR 0.58, 95% CI 0.45–0.75; *p* < 0.001). After a median follow up time of 18.8 months, the other coprimary endpoint was also achieved with a median OS of 21.4 months (95% CI 18.7–not estimable) with cabozantinib versus 16.5 months (14.7–18.8) with everolimus (HR 0.66 (95% CI 0.53–0.83); *p* = 0.00026) [109]. Disease control rate was again a strength for cabozantinib reaching the 82%.

The development of cabozantinib has reached the first line setting in the phase II CABOSUN trial [101]. This study was conducted by the Alliance for Clinical Trials in Oncology study and randomized 157 patients belonging to the intermediate (81% patients) and poor (19% of patients) IMDC risk group prognosis to cabozantinib versus sunitinib at the standard dose regimens. Particularly in this trial, 13% of patients were Eastern Cooperative Oncology Group Performance Status (ECOG PS) of 2 and 37% of patients had bone metastases. The primary endpoint of PFS assessed by investigator was 8.6 months for cabozantinib versus 5.3 months for sunitinib (0.66; 95% CI, 0.46 to 0.95; one-sided *p* = 0.012). After a median follow up of 25 months (range 21.9–30.9) updated results were presented (after 92 events for PFS): HR for PFS was 0.56 (95% CI 0.37–0.83; *p* = 0.0042) and the ORR was 20% for cabozantinib versus 9% for sunitinib by blinded independent-central review (BICR) and 33% for cabozantinib versus 12% for sunitinib by investigator assessment. This trial included the immunohistochemical analysis of MET in tumor tissue, available for 131 patients. Approximately 50% of patients showed MET expression and a greater probability of benefit from cabozantinib treatment (median PFS = 13.8 months for cabozantinib versus 3.0 months for sunitinib (HR 0.32; 95% CI 0.16–0.63)) [102].

### 4.6. Lenvatinib

The activity of lenvatinib is based on the activity over FGFR inhibition related to resistant mechanisms to classic therapeutic inhibition of VEGFR. The role in kidney cancer is based on the phase II HOPE 205 that randomized 153 patients progressing to the previous treatment with one VEGFR targeted agent to three different arms: Lenvatinib 24 mg/day (*N* = 52) versus lenvatinib plus everolimus 5 mg/day (*N* = 51) versus everolimus 10 mg/day (*N* = 50) [110] (Table 2). Both arms containing lenvatinib improved the median PFS over everolimus (mPFS lenvatinib plus everolimus versus everolimus = 14.6 months versus 5.5 months (HR 0.40, 95% CI 0.24–0.68; *p* = 0.0005) and median PFS lenvatinib versus everolimus = 7.4 months versus 5.5 months (HR 0.61, 95% CI 0.38–0.98; *p* = 0.048)). Those promising results led to the approval of lenvatinib for second line treatment and the development of the phase III CLEAR trial currently recruiting patients (NCT02811861) (Table 3).

### 4.7. Sorafenib

The role of VEGFR TKI was firstly demonstrated based on preclinical activity data in VHL-knockout murine models and, consequently, in a phase II trial including patients with metastatic ccRCC. The activity sorafenib is based on its ability to act over VEGFR 1-3, PDGFRβ, Flt-3, c-Kit and RET receptor tyrosine kinases. Although compared with the placebo, sorafenib 400 mg twice daily demonstrated a survival benefit in patients with favorable and intermediate-risk MSKCC criteria metastatic ccRCC previously treated with at least one systemic treatment (cytokine-based therapy) [111]. The primary endpoint of OS was achieved at the first analysis and after the allowance of the crossover of patients from the placebo arm to sorafenib treatment. During the following years, sorafenib has been considered the control arm in different clinical trials in the first and subsequent treatment lines [99,102,112]. 

## 5. The Impact of Immunotherapy in an Angiogenic Disease

The involvement of VHL in kidney cancer was the first hit that allowed the development of all the previously mentioned TKIs treatments leading a historic change in metastatic RCC survival. Moreover, the immune system has also been investigated as a relevant player in kidney cancer behaviour and target agents have demonstrated survival impact in clinical trials with novel immune-based therapies. Indeed, the benefit in OS was reached by the Checkmate 025 trial where patients in the second- and third-line treatment were randomized to receive nivolumab versus everolimus [113]. The primary endpoint was achieved (median OS = 25 months for nivolumab versus 19.6 months for everolimus (HR = 0.73, *p* = 0.002)) and nivolumab became a new standard of care after treatment with a VEGFR inhibitor. Furthermore, the double immunotherapy combination (PD1/CTLA4) was evaluated in the first line setting in the Checkmate 214 trial including 1096 patients that were randomized to receive nivolumab 3 mg/kg plus ipilimumab 1 mg/kg versus sunitinib at standard dose [10] (Table 3). The results showed a significant benefit in the coprimary endpoints of ORR (42% with nivolumab plus ipilimumab versus 27% with sunitinib, *p* < 0.001), median PFS (11.6 months with nivolumab plus ipilimumab and 8.4 months with sunitinib, HR = 0.82, *p* = 0.03) and median OS (not reached with nivolumab plus ipilimumab versus 26.0 months with sunitinib, HR = 0.63, *p* < 0.001), among intermediate- and poor-risk patients. After these results, the double immunotherapy combination was approved in this setting. Even though it was not considered a primary endpoint of the trial, interesting results were reported from the favorable risk MSKCC group. In this exploratory analysis, the ORR was 29% (11% of complete responses) with nivolumab plus ipilimumab versus 52% (6% of complete responses) with sunitinib. The median PFS in this subgroup was 15.3 months with nivolumab plus ipilimumab versus 25.1 months with sunitinib and the median OS was not reached with nivolumab plus ipilimumab compared with 32.9 months in the sunitinib arm. The updated results of the coprimary endpoints presented in the 2019 Genitourinary Cancers Symposium after a minimum follow up of 30 months (median 32.4 months) still show a benefit of nivolumab plus ipilimumab in the Intention-To-Treat (ITT) and intermediate- and poor-risk patients [114]. On the contrary, in the favourable-risk group, no significant benefit was reported.

Remarkably, immunotherapy has also been combined with TKIs in the first line setting of kidney cancer. This treatment strategy is currently being evaluated in different phase III trials, two of them have recently published their results [115,116] (Table 3). The first one is the MK426 trial that randomized 861 patients with metastatic ccRCC to receive treatment with pembrolizumab 200 mg plus axitinib 5 mg/12 h versus sunitinib 50 mg/24 h four weeks on/two weeks off [115]. After a median follow-up of 12.8 months, the study met its primary endpoints with a median OS not reached in both groups (HR = 0.53; *p* < 0.0001) and a median PFS of 15.1 months with pembrolizumab plus axitinib versus 11.1 months with sunitinib (HR = 0.69; *p* < 0.001). The ORR was 59.3% (complete responses = 5.8%) with pembrolizumab plus axitinib and 35.7% (complete responses = 1.9%) with sunitinib. The second trial is the JAVELIN RENAL 101 that randomized 886 patients to receive avelumab 10 mg/kg plus axitinib 5 mg/12 h versus sunitinib 50 mg/24 h four weeks on/two weeks off [116]. The study also met its coprimary endpoints of PFS and OS among patients with PDL-1 positive tumors. A PDL1-positive tumor was found in 560 patients (63.2%) and, after a median follow up of 11.6 months, the median PFS was 13.8 months with avelumab plus axitinib versus 7.2 months with sunitinib (HR = 0.61; *p* < 0.001) and data on OS were still immature due to the low number of events (37 patients had died in the avelumab plus axitinib group and 44 patients had died in the sunitinib group).

## 6. Discussion: Considerations in RCC Treatment

Since almost the last 15 years, the background of TKIs in kidney cancer has progressively increased from preclinical data to the TKI sequences that have been able to offer our patients, at each time, a new opportunity of response and improvement in survival. From the first TKIs developed to the latest ones, the ability of one TKI to induce response have not prevented from the following one to re-induce another tumor response and, consequently, have an impact on patient survival. This characteristic is based on the different sensitivities of TKIs to act over the tyrosine kinases overexpressed in kidney cancer [90]. In fact, the currently approved TKIs in kidney cancer have demonstrated activity in different mechanisms of tumor growth, progression and resistance to therapeutic pressure. This different activity over VEGFR 1 to 3, PDGFR, Axl, FGFR or MET allows a treatment algorithm with TKIs sequences.

Indeed, this therapeutic benefit has spread to other tumor types, such as thyroid [118,119], hepatocarcinoma [120] or neuroendocrine tumors [121,122].

To date, treatment sequencing with TKIs has generally pursued two main objectives according to the little benefit obtained from cytokine-based therapy: Survival and safety profile. However, with the increase in survival and improvement in treatment-related toxicity, other goals have been demanded. In this sense, quality of life has become increasingly more important in clinical trials and patients are routinely asked for this endpoint during treatment period. Furthermore, the increase in tumor size reduction reported in the phase III clinical trials from the double immunotherapy or the TKIs plus immunotherapy combos, would modify the current approach of surgical or other local strategies (upfront versus deferred) in the advanced disease setting. Indeed, novel TKIs with a greater ability of tumor shrinkage, such as cabozantinib, are being evaluated in other clinical settings, such as the perioperative treatment in the CABOPRE trial, as well as immune checkpoint inhibitors, such as nivolumab, in the PROSPER trial (NCT03055013). Indeed, primary tumor resection in patients with metastatic RCC, called cytoreductive nephrectomy, takes part in the multidisciplinary therapeutic algorithm of kidney cancer. The paradigm of cytoreductive nephrectomy has been related to the benefit in survival in patients treated with cytokine-based therapy. This surgical approach also pursues tumor-related symptoms relief and avoidance of potential local complications derived from the primary tumor growth (pain, bleed or infection, among others). Patients with a good performance status, absence of metastases in unfavorable locations (liver, bone or central nervous system) and with most of tumor burden within the primary tumor, have been considered the best candidates for cytoreductive nephrectomy [123]. However, when TKIs came to the frontline of metastatic RCC treatment, the debate about the best patient selection criteria arouse again and the results of the recently published CARMENA trial evaluating the benefit of the upfront cytoreductive nephrectomy followed by sunitinib versus sunitinib alone, confirmed the current clinical feeling about the necessity of a careful and multidisciplinary patient selection for this multimodal strategy [124,125].

Even though TKIs and immunotherapy, separately, have demonstrated a benefit in response rate and survival, metastatic RCC is still a deadly disease and novel therapeutic targets are trying to increase the number of patients reaching a complete response to approach the “diamond age” of kidney cancer [2]. The results obtained in the trials that evaluate the double immunotherapy (Checkmate 214) and the TKI plus immunotherapy combos (MK426 and JAVELIN RENAL 101) are undeniable and it is expected that both, pembrolizumab plus axitinib and avelumab plus axitinib, also become a standard of care endorsed by clinical guidelines in the first line setting together with nivolumab plus ipilimumab. However, there are some important issues in the future management of metastatic kidney cancer that those phase III trials, though showing better results, would not be able to answer. First of all, those clinical trials have all considered sunitinib as the comparator arm [126]. This strategy will make it difficult to interpret the results as TKIs considered in the combination arms are different from sunitinib, such as axitinib, cabozantinib or lenvatinib. In fact, only the CLEAR trial (NCT02811861) has a third arm, that in this case includes the combination of lenvatinib plus everolimus. However, the rest of these trials lack of an additional arm with the combined TKI and/or the immunotherapeutic agent alone. This consideration would have allowed a direct comparison with sunitinib and with the combination arm, and more reliable results for definitive conclusions. Moreover, population in those trials is of course different, so the assumption of one trial better than the other would not be possible. For example, the MK426 trial included almost 30% of patients with favorable prognosis criteria, compared with the JAVELIN RENAL 101 (21%) and Checkmate 214 (23%). In addition, the pre-specified endpoints are different among these trials and there are different statistical considerations, i.e., the JAVELIN RENAL 101 primarily evaluates the PDL1-positive tumor population, the MK426 does the same in the ITT population and the Checkmate 214 in the intermediate- and poor-risk MSKCC prognostic group population.

The upcoming change in the first line setting will result in a consequent modification of the subsequent treatment lines according to the schedule administered upfront. However, only retrospective data and exploratory analysis are currently available to justify the activity of TKIs after immune based therapy. In this sense, ongoing clinical trials are evaluating the activity of TKIs after the administration of a single/double immunotherapy or a TKI and immunotherapy combination. This is the case of pazopanib (IO-PAZ; NCT03200717) sunitinib (INMUNOSUN; NCT03066427) or cabozantinib (BREAKPOINT; NCT03463681). Those trials will improve the evidence-based efficacy about the activity of TKIs after those novel combos.

Finally, research is going further in kidney cancer, but new strategies, different from TKIs and immune-checkpoint inhibitors, have not currently achieved phase III trials. In this sense, alternative proangiogenic pathways that are involved in resistance mechanisms to VEGFR inhibitors have been evaluated, such as angiopoietin inhibitors [127,128] or HIF-2α inhibitors [129]. Drugs involved in metabolism pathways are also being evaluated in phase II clinical trials, such as CB-839, a glutaminase inhibitor that in combination with cabozantinib achieves an ORR of 42% [130]. Those promising results have led the development of the currently recruiting randomized phase II trial CANTATA (NCT03428217). Otherwise, Bruton’s tyrosine kinase (BTK), as a key intracellular signaling both for B and T lymphocytes, is under current research in combination with nivolumab (NCT02899078) and everolimus (NCT02599324) [131]. Finally, other relevant investigational strategies that are arising promising results are based on histone deacetylase (NCT02619253, NCT03024437) [132], BET proteins (NCT02419417) or NOTCH inhibitors (NCT01198184). Special attention should be given to the role of non-coding RNAs as key mechanisms in ccRCC development and progression, accordingly with the importance of epigenetic alterations in this oncological context. In fact, several reports have identified microRNAs (miRNAs) associated to ccRCC stages [133]. New therapies based on miRNAs modulations could contribute to the angiogenesis control in ccRCC as it has been already suggested [134], by themselves or in combination with the currently available anti-angiogenic drugs widely discussed in this review.

## 7. Conclusions

The preclinical knowledge about the key role of tyrosine kinases in kidney cancer development has driven the approval of different TKIs that have demonstrated significant benefit in survival of patients with metastatic RCC. The efficacy demonstrated and the manageable safety profile have allowed the inclusion of this therapeutic strategy as the cornerstone of metastatic RCC treatment sequencing and, though the novel immunotherapeutic agents approved in kidney cancer, a required partner in the front line setting in combination with immune checkpoint inhibitors.

## Figures and Tables

**Table 1:** Tyrosine kinase inhibitors (TKIs) evaluated in phase II and III clinical trials in the first line setting. † Updated analysis, after a median follow up of 34.5 months.

TKIs	Sunitinib [97]	Pazopanib COMPARZ [98]	Tivozanib TIVO-1 [99]	Cabozantinib CABOSUN [101,102]
Treatment line	1^st^	1^st^	1^st^	1^st^
Study design	Phase III	Phase III Non inferiority	Phase III	Phase II
*N*	750	1110	517	157
Comparator arm	IFN α	Sunitinib	Sorafenib	Sunitinib
ORR (%)	31 vs. 6	31 vs. 25	33.1 vs. 23.4	20 vs. 9
PFS (months)	11 vs. 5 (HR 0.42; *p* < 0.001)	8.4 vs. 9.5 (HR 1.05)	11.9 vs. 9.1 (HR 0.79; *p* = 0.042)	8.6 vs. 5.3 (HR 0.66; *p* = 0.012)
OS (months)	26.4 vs. 21.8 (HR 0.82)	28.4 vs. 29.3 (HR 0.91)	29.3 vs. 28.8 (HR 1.24; *p* = 0.105)	26.6 vs. 21.2 (HR 0.80) †
Adverse events	Hypertension, Diarrhea, Fatigue, HFS, Leukopenia, Thrombocytopenia	Fatigue, PPE AST, ALT, Br increase	Hypertension, Dysphonia	Hypertension, Diarrhea, Anorexia, PPE, Weight loss
Approval regulatory authorities	2006 (FDA) 2006 (EMA)	2009 (FDA) 2010 (EMA)	2017 (EMA)	2017 (FDA) 2018 (EMA)

**Table 2:** TKIs evaluated in phase II and III clinical trials in patients that have received previous treatment. * Interim analysis. # PPE: Palmar-plantar erythrodysaesthesia. † Updated results (cutoff date December 10th, 2014). ‡ After a median follow up of 6.6 months. ‡‡ After 47.7% of patients in the placebo arm had switched to sorafenib.

TKIs	Axitinib AXIS [102]	Cabozantinib METEOR [106,107]	Lenvatinib + Everolimus HOPE 205 [110]	Tivozanib TIVO-3 [101]	Sorafenib [111]
Treatment line	2^nd^	≥2^nd^	2^nd^	3^rd^ and 4^th^	≥2^nd^ cytokine-based therapy
*N*	723	375	153	350	903
Comparator arm	Sorafenib	Everolimus	Lenvatinib vs. Everolimus	Sorafenib	Placebo
ORR (%)	19 vs. 9	17 vs. 3	30 vs. 19 vs. 20	18 vs. 8	44 vs. 2
PFS (months)	6.7 vs. 4.7 (HR 0.665; *p* = 0.0001)	7.4 vs. 3.8 (HR 0.58; *p* < 0.001)	14.6 vs. 7.4 vs. 5.5 (HR 0.4; *p* = 0.0005 and HR 0.6; *p* = 0.048)	5.6 vs. 3.9 (HR 0.73; *p* = 0.0165)	5.5 vs. 2.8 (HR 0.44; *p* < 0.001)
OS (months)	20.1 vs. 19.2 (HR 0.97; *p* = 0.37)	21.4 vs. 16.5 (HR 0.66; *p* = 0.0003)	25.5 vs. 19.1 vs. 15.4 (HR 0.51; *p* = 0.024 and HR 0.68; *p* = 0.12) †	16.4 vs. 19.7 (HR 1.12; *p* = 0.4) *	NR vs. 14.7 (HR 0.72; *p* = 0.02) ‡ 19.3 vs. 15.9 (HR 0.77; *p* = 0.02) ‡‡
Adverse events	Hypertension, Diarrhea, Fatigue, Anorexia, Asthenia, PPE#	Hypertension, Diarrhea, Fatigue, PPE#, anaemia, ionic disorders	Diarrhea, Hypertension, Fatigue, Anorexia, Proteinuria, Hypertrygliceridaemia, Nausea/Vomiting, Decreased weight, Hyperglycaemia, Dyspnoea	Hypertension, Fatigue, Diarrhea, Anorexia, Dysphonia	PPE, Fatigue, Hypertension, Anaemia, Dyspnea, Diarrhea
Approval regulatory authorities	2012 (FDA) 2012 (EMA)	FDA 2016 EMA 2016	FDA 2016 EMA 2016	-	2005 (FDA)

**Table 3:** Recently approved combinations and upcoming drugs in the first line treatment for patients with metastatic renal cell carcinoma (RCC) combining VEGF/R and PD1/PDL1 inhibition. NR: Not reached; NRe: Not Reported.

Treatment and Study	Checkmate 214 Nivolumab + Ipilimumab [10,114]	MK426 Pembrolizumab + Axitinib [115]	IMmotion 151 Atezolizumab +bevacizumab [117]	JAVELIN RENAL 101 Avelumab + Axitinib [116]	CLEAR Pembrolizumab + Lenvatinib	CheckMate 9ER Nivolumab + Cabozantinib	Tivozanib + Nivolumab
Comparator arm	Sunitinib	Sunitinib	Sunitinb	Sunitinib	Sunitinib	Sunitinib	-
Primary endpoint	ORR PFS OS	PFS OS	PFS (Investigator) PDL1+ OS ITT	PFS PDL1ve OS PDL1ve	PFS	PFS	Safety/security
Results initially presented	ESMO 2017	ASCO GU 2019	ASCO GU 2018	ESMO 2018	Recruiting	Recruiting	Recruiting
Median Follow Up (months)	32.4	12.8	15	9.9	NRe	NRe	NRe
ORR (%)	42	59.3	43	55.2	NRe	NRe	NRe
PFS (months)	11.6	15.2	11.2	13.8	NRe	NRe	NRe
mOS (months)	NR	NR	NRe	NR	NRe	NRe	NRe

## Appendix A

Memorial Sloan-Kettering Cancer Center (MSKCC/Motzer) Score for Metastatic RCC classified the patients according to five items in favorable, intermediate and poor risk. Each of these groups have different survival outcomes. Patients with 0 points were included in the favorable risk group and achieved a median OS of 30 months. Patients with 1-2 points were included in the intermediate risk group and achieved a median OS of 14 months. Patients with more than 2 points were included in the poor risk group and achieved a median OS of five months [91].

	**Yes (points)**	**No (points)**
**Hemoglobin < Lower Limit of Normal**	**1**	**0**
**Time from diagnosis to systemic treatment <1 year**	**1**	**0**
**Calcium >10mg/dL (>2.5 mmol/L)**	**1**	**0**
**Performance status <80% (Karnofsky)**	**1**	**0**
**LDH > 1.5x Upper Limit of Normal**	**1**	**0**

## Appendix B

IMDC (International Metastatic RCC Database Consortium) Risk Score for metastatic RCC classified the patients according to six items in favorable, intermediate and poor risk. Each of this groups have different survival outcomes. Patients with 0 points were included in the favorable risk group and median OS was not reached (2-year OS was 75%). Patients with 1-2 points were included in the intermediate risk group and achieved a median OS of 27 months. Patients with more than 2 points were included in the poor risk group and achieved a median OS of 8.8 months [92].

	**Yes (points)**	**No (points)**
**Performance status <80% (Karnofsky)**	**1**	**0**
**Less than one year from time of diagnosis to systemic therapy**	**1**	**0**
**Calcium > upper limit of normal**	**1**	**0**
**Hemoglobin < lower limit of normal**	**1**	**0**
**Neutrophils > upper limit of normal**	**1**	**0**
**Platelets > upper limit of normal**	**1**	**0**
